# The Longitudinal Associations of Denture Use and Subjective Chewing Difficulty with Depressive Symptoms Among Middle-Aged and Older Adults: A Sex-Stratified Analysis of the Korean Longitudinal Study of Aging (2006–2022)

**DOI:** 10.3390/healthcare14182912

**Published:** 2026-09-09

**Authors:** Myeong-Hwa Park, Jae-Hyun Kim, Jong-Hwa Jang

**Affiliations:** 1 Department of Public Health Sciences, Dankook University Graduate School, Cheonan-si 31116, Republic of Korea; myeonghwa.park.ln@army.mil; 2 Institute for Oral Health and Integrated Care, Dankook University, Cheonan-si 31116, Republic of Korea; 3 Department of Health Administration, College of Health Sciences, Dankook University, Cheonan-si 31116, Republic of Korea; jaehyun@dankook.ac.kr; 4 Department of Dental Hygiene, College of Health Sciences, Dankook University, Cheonan-si 31116, Republic of Korea

**Keywords:** depressive symptoms, subjective chewing difficulty, mastication, dentures, middle-aged, aged, sex factors, Korean Longitudinal Study of Aging

## Abstract

**Highlights:**

**What are the main findings?**

- Greater subjective chewing difficulty was consistently associated with higher depressive symptom scores across repeated observations among both denture users and non-denture users.
- Formal interaction analyses indicated that sex modified the associations of denture use and subjective chewing difficulty with depressive symptoms. The association of denture use with depressive symptoms was observed among women but not among men, while the associations between chewing difficulty and depressive symptoms differed by sex in both denture status groups.

**What are the implications of the main findings?**

- Perceived chewing function may provide additional information beyond denture status when assessing oral health-related vulnerability associated with depressive symptoms.
- Oral function and mental health may warrant joint consideration when evaluating middle-aged and older adults, although the temporal direction of these associations requires further investigation.

**Abstract:**

**Background/Objectives:** Evidence from repeated-measures studies examining denture use, subjective chewing difficulty, and depressive symptoms remains limited. This study examined the population-averaged associations of denture use and subjective chewing difficulty with depressive symptoms across nine waves of the Korean Longitudinal Study of Aging (KLoSA). **Methods:** Data from 10,137 participants contributing 68,937 person-wave observations across nine KLoSA waves (2006–2022) were analyzed. Depressive symptoms were assessed using the 10-item Center for Epidemiologic Studies Depression Scale (CESD-10). Generalized estimating equations (GEEs) were used to account for repeated observations within participants. Sex-stratified analyses and formal interaction tests were additionally performed. **Results:** In adjusted sex-stratified analyses, denture use was associated with a higher expected mean CESD-10 score among women [exp(B) = 1.068, 95% CI: 1.044–1.092] but not among men. Greater subjective chewing difficulty was consistently associated with higher expected mean CESD-10 scores among both men and women and among both denture users and non-denture users. The sex × denture use interaction was significant [exp(B) = 1.064, 95% CI: 1.043–1.085, *p* < 0.001], and sex × subjective chewing difficulty interactions were also significant among both denture users and non-denture users (all *p* ≤ 0.001). **Conclusions:** Greater subjective chewing difficulty was consistently associated with higher depressive symptom levels across repeated observations among both denture users and non-denture users. Sex modified the associations of denture use and subjective chewing difficulty with depressive symptoms. Perceived chewing function may provide additional information beyond denture status when assessing oral health-related vulnerability associated with depressive symptoms; however, temporal direction and causality cannot be inferred from these analyses.

## 1. Introduction

Korea became a super-aged society at the end of 2024, with adults aged ≥ 65 years accounting for more than 20% of the registered population [1]. Accordingly, maintaining physical, mental, and social functioning among middle-aged and older adults has become increasingly important. Oral health is a key component of healthy aging because of its close relationship with nutritional status, physical and cognitive functions, mental health, and quality of life [2].

Masticatory function is essential for food comminution and bolus preparation and contributes to dietary intake, swallowing, and nutritional status in older adults [3,4]. Impaired mastication and chewing difficulty have been associated with malnutrition, sarcopenia, frailty, aspiration pneumonia, and functional decline [5,6,7,8,9]. Chewing difficulty may also reduce dietary variety and eating enjoyment, limit participation in social meals, and contribute to loneliness and depressive symptoms. Recent longitudinal evidence suggests that loneliness may partially mediate the association between chewing disability and depressive symptoms [5,6]. Mastication may also support cognitive function through sensory stimulation and activation of neural pathways involved in memory and executive function, although the underlying mechanisms and direction of causality remain uncertain [3].

Previous studies have reported associations between prosthetic status, impaired masticatory function, and depressive symptoms [10]. Additionally, studies using the Korean Longitudinal Study of Aging (KLoSA) have identified relationships among denture use, oral health-related quality of life, sensory impairment, depressive symptoms, cognitive function, and quality of life [11,12]. Nevertheless, evidence remains limited regarding the associations of denture use and subjective chewing difficulty with depressive symptoms across repeated observations. In addition, whether these associations differ by sex has not been sufficiently examined, despite potential sex differences in biological characteristics, health behaviors, social roles, social support, and psychological vulnerability [13,14,15,16,17].

This study used nine waves of the KLoSA to examine the associations of denture use and subjective chewing difficulty with depressive symptoms across repeated observations. We hypothesized that greater subjective chewing difficulty would be associated with higher depressive symptom levels and that these associations would differ by sex.

## 2. Materials and Methods

### 2.1. Study Design, Data Source, and Population

This study was a secondary analysis of publicly available longitudinal panel data from nine waves of the KLoSA: 2006, 2008, 2010, 2012, 2014, 2016, 2018, 2020, and 2022. KLoSA is a nationally representative longitudinal panel survey administered by the Korea Employment Information Service using multistage stratified systematic sampling based on geographic region and housing type [18]. Trained interviewers collected data through computer-assisted personal interviews [19].

**Figure 1:**
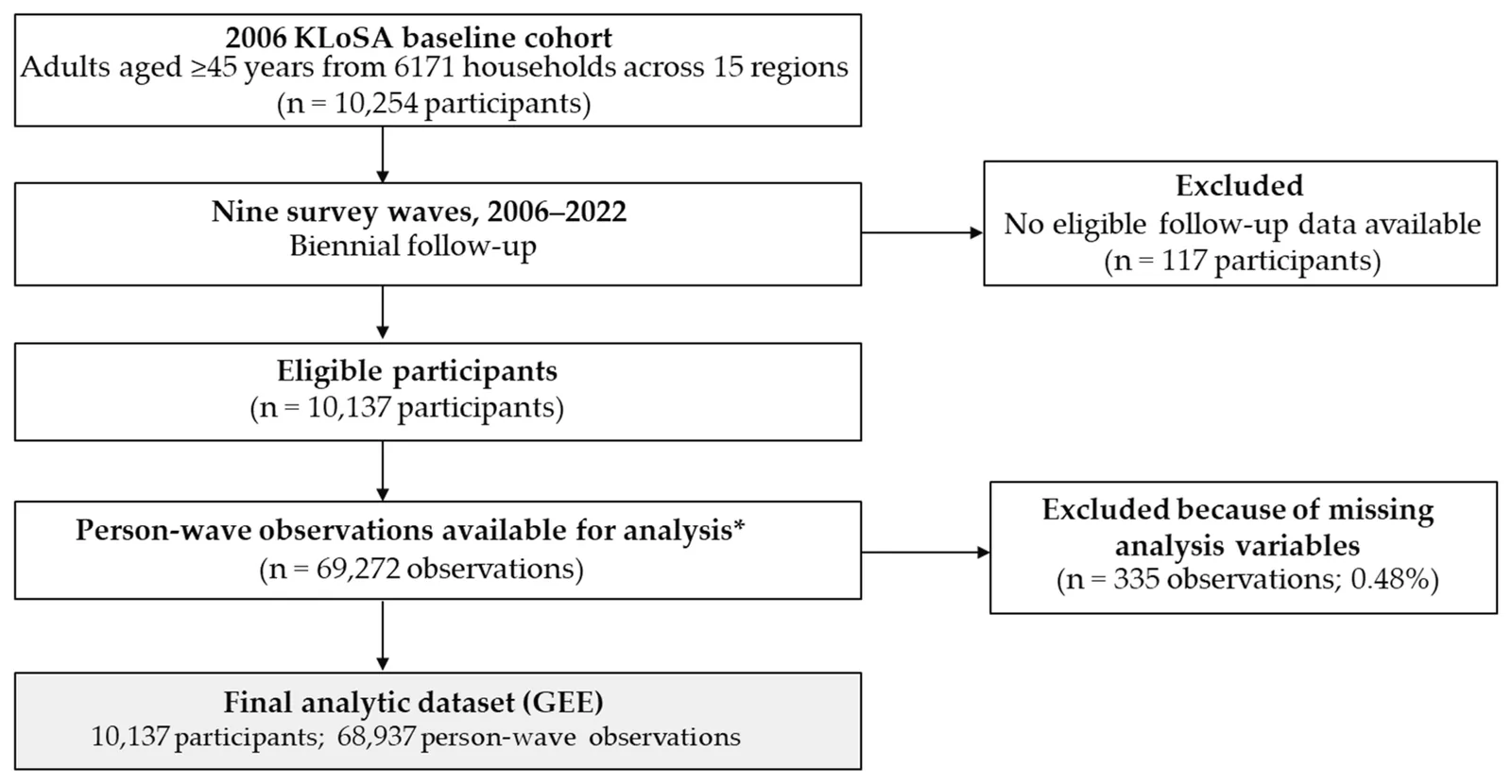
A flowchart of participant selection from the Korean Longitudinal Study of Aging (KLoSA), 2006–2022. Person-wave observations with missing data for the analysis variables (n = 335): CESD-10 score (n = 266); health insurance status (n = 23); chronic disease count (n = 12); marital status (n = 1); and other variables (n = 33). * Person-wave observations represent all available interviews contributed by 10,137 participants across nine waves. Other variables included age, sex, education level, activity limitation, and alcohol consumption. GEE, generalized estimating equations; CESD-10, 10-item Center for Epidemiologic Studies Depression Scale.

The baseline cohort consisted of 10,254 participants. After applying the eligibility criteria, 10,137 unique participants contributed data to the repeated-measures analyses. Because the number of available observations differed across survey waves owing to loss to follow-up, death, non-response, and item-level missingness, the GEE models were based on available repeated observations and included a total of 68,937 person-wave observations. The overall participant flow and reasons for exclusion are presented in Figure 1, while the numbers of participants contributing observations at each survey wave are provided in Supplementary Table S1.

### 2.2. Measures

#### 2.2.1. Denture Use and Subjective Chewing Difficulty

Denture use was classified as “yes” or “no” according to participants’ responses to the question “Do you usually wear dentures?”. Subjective chewing difficulty was assessed using denture status-specific questions [7,20]. Denture users were asked whether they could bite and chew hard foods such as apples or meat without difficulty while wearing their dentures. Participants who did not use dentures were asked whether they could bite and chew such foods without dentures.

Responses to each question were classified into five categories: “very well,” “well,” “moderately,” “with difficulty,” and “unable to chew at all.” Responses were coded from 1 (“very well”) to 5 (“unable to chew at all”), with higher scores indicating greater subjective chewing difficulty. Because the questions were administered under different functional contexts, chewing difficulty among denture users reflects perceived prosthesis-assisted chewing ability, whereas chewing difficulty among non-users reflects perceived chewing ability without dentures.

#### 2.2.2. Depressive Symptoms

Depressive symptoms were assessed using the 10-item Center for Epidemiologic Studies Depression Scale (CESD-10) [21,22], which consists of 10 items assessing the frequency of depressive symptoms experienced during the previous week. Each item was scored 0 when the symptom was experienced for less than 1 day or 1–2 days and 1 when it was experienced for 3–4 days or 5–7 days. Positively worded items were reverse-scored, and the item scores were summed to calculate the total score. Total scores ranged from 0 to 10, with higher scores indicating greater depressive symptom levels.

#### 2.2.3. Covariates

Potential confounders that could influence the associations of denture use and subjective chewing difficulty with depressive symptoms were selected based on previous studies and included sociodemographic and health-related characteristics [6,23]. Except for sex and educational level, covariates were treated as time-varying variables using values measured at each survey wave.

Sociodemographic characteristics included age, sex, educational level, and marital status. Age was categorized as ≤54, 55–64, 65–74, and ≥75 years. Educational level was classified as elementary school or lower, middle school, high school, and college or higher. Marital status was categorized according to the response categories in the original dataset: married, divorced or separated, and never married.

Health-related characteristics included activity limitation, alcohol consumption, health insurance type, and number of chronic conditions. Activity limitation and alcohol consumption were each classified as “yes” or “no”. Health insurance type was categorized as National Health Insurance or Medical Aid. The number of chronic conditions was calculated by assigning one point for each physician-diagnosed condition, including hypertension, diabetes mellitus, cancer, chronic obstructive pulmonary disease, liver disease, cardiovascular disease, cerebrovascular disease, psychiatric disorders, and arthritis. The total number of chronic conditions was subsequently categorized as 0, 1, or ≥2 for analysis.

### 2.3. Statistical Analysis and Sensitivity Analysis

Participants’ general characteristics and CESD-10 scores according to these characteristics were examined for the total sample and separately by sex. Categorical characteristics are presented as frequencies and percentages, and CESD-10 scores are presented as means and standard deviations. Differences in mean CESD-10 scores across categories of participant characteristics were assessed using independent *t*-tests for variables with two categories and one-way analysis of variance (ANOVA) for variables with three or more categories.

Generalized estimating equations (GEEs) were used to estimate population-averaged associations of denture use and subjective chewing difficulty with CESD-10 scores across repeated observations within individuals. The participant-specific unique identifier (PID) was specified as the clustering variable. A normal distribution with a log link function was specified for CESD-10 scores, and an independent working correlation structure was assumed for repeated observations. Robust (sandwich) standard errors were used to account for potential misspecification of the working correlation structure. Because a log link function was used, exponentiated regression coefficients [exp(B)] were reported and interpreted as ratios of expected mean CESD-10 scores relative to the corresponding reference category.

Unequal numbers of repeated observations across participants were permitted. For each model, all available observations with complete information on the variables required for that analysis were included. Because the missing-data mechanism could not be determined from the available data, potential bias due to missing observations and informative attrition was considered a study limitation. The GEE analyses were conducted without incorporating survey weights or strata; accordingly, the resulting estimates represent unweighted population-averaged associations within the analytic cohort rather than nationally weighted population estimates.

Except for sex and educational level, covariates were treated as time-varying and were updated at each survey wave. Survey year was included as a categorical covariate to account flexibly for wave-specific differences without assuming a linear temporal trend, with 2022 as the reference category. Multivariable models were adjusted for age, educational level, marital status, activity limitation, alcohol consumption, health insurance type, and the number of chronic conditions.

In addition to sex-stratified analyses, formal interaction analyses were performed using GEE models. The sex × denture use interaction was tested in the pooled sample, whereas sex × subjective chewing difficulty interactions were tested separately among denture users and non-denture users. Exponentiated regression coefficients [exp(B)], 95% confidence intervals (CIs), and *p*-values were reported. 

As a sensitivity analysis, subjective chewing difficulty was recategorized into three levels: Good (“very well” and “well”), Moderate (“moderately”), and Poor (“with difficulty” and “unable to chew at all”). The GEE analyses were repeated using the same distribution, link function, working correlation structure, and covariate adjustments as in the primary five-category analyses to assess the robustness of the findings to alternative categorization of subjective chewing difficulty and the relatively small number of observations in the most severe category. The sex-stratified associations based on the three-level classification are presented in Supplementary Table S2. Additional sex × chewing difficulty interaction analyses using the recategorized variable are presented in Supplementary Table S3.

All analyses were performed using SAS version 9.4 (SAS Institute Inc., Cary, NC, USA). All statistical tests were two-sided, and statistical significance was set at α = 0.05.

### 2.4. Ethical Considerations

This study used publicly available, de-identified secondary data from the KLoSA. The study protocol was reviewed by the Institutional Review Board (IRB) of Dankook University and was determined to be exempt from further ethical review (IRB No. DKU 2025-05-013; 28 May 2025). The study was conducted in accordance with the Declaration of Helsinki and was reported in accordance with the Strengthening the Reporting of Observational Studies in Epidemiology (STROBE) guidelines. All participants provided informed consent during the original KLoSA data collection process.

## 3. Results

### 3.1. Depressive Symptom Levels According to the General Characteristics, Stratified by Sex

Table 1 presents the participants’ baseline characteristics in 2006 and CESD-10 scores stratified by sex. Women accounted for 56.4% of the participants and had significantly higher CESD-10 scores than men (*p* < 0.001).

CESD-10 scores differed significantly across age groups, educational levels, marital-status categories, and activity-limitation status (all *p* < 0.001). Medical Aid beneficiaries also had higher CESD-10 scores than National Health Insurance beneficiaries. CESD-10 scores also differed significantly according to the number of chronic diseases (*p* < 0.001).

Alcohol consumption was not significantly associated with CESD-10 scores in the overall sample (*p* = 0.208). However, among women, drinkers had significantly higher scores than non-drinkers (*p* < 0.001).

### 3.2. Depressive Symptoms According to Subjective Chewing Difficulty, Stratified by Sex and Denture Use

Table 2 presents CESD-10 scores according to denture use and subjective chewing difficulty, stratified by sex. The mean CESD-10 scores differed significantly between denture users and non-users among both men and women (all *p* < 0.001). Regardless of denture use, CESD-10 scores differed significantly across levels of subjective chewing difficulty, with the highest mean scores observed among participants who were unable to chew at all (all *p* < 0.001).

### 3.3. Association Between Denture Use and Depressive Symptoms by Sex

Table 3 presents the sex-stratified association between denture use and depressive symptoms across repeated observations. After adjustment for covariates, denture use was not significantly associated with the expected mean CESD-10 score among men [exp(B) = 1.018, 95% CI: 0.991–1.045, *p* = 0.203]. However, among women, denture users had an approximately 6.8% higher expected mean CESD-10 score than non-users [exp(B) = 1.068, 95% CI: 1.044–1.092, *p* < 0.001].

Older age, activity limitation, Medical Aid coverage, and a greater number of chronic conditions were associated with higher expected mean CESD-10 scores. Lower educational attainment was significantly associated with higher expected mean scores only among men. Compared with married participants, divorced or separated men had higher expected mean CESD-10 scores, while never-married status was additionally associated with higher expected mean scores among women. CESD-10 scores also varied across survey years, although the pattern differed by sex.

### 3.4. Association Between Subjective Chewing Difficulty and Depressive Symptoms, Stratified by Sex and Denture Use

Table 4 presents the association between subjective chewing difficulty and depressive symptoms across repeated observations, stratified by sex and denture status. Among denture users, greater subjective chewing difficulty was significantly associated with higher expected mean CESD-10 scores in both men and women. Compared with participants who could chew very well, those who were unable to chew at all had higher expected mean CESD-10 scores among both men [exp(B) = 1.911, 95% CI: 1.418–2.576] and women [exp(B) = 1.875, 95% CI: 1.297–2.712; both *p* < 0.001].

Similar associations were observed among non-denture users. Compared with participants who could chew very well, those who were unable to chew at all had higher expected mean CESD-10 scores among both men [exp(B) = 1.553, 95% CI: 1.403–1.720] and women [exp(B) = 1.648, 95% CI: 1.500–1.811; both *p* < 0.001].

Activity limitation and a greater number of chronic conditions were consistently associated with higher expected mean CESD-10 scores. Associations with age, marital status, alcohol consumption, and health insurance type varied across the models stratified by sex and denture status. Survey-year estimates also varied across models; however, these estimates represent relative differences in expected mean CESD-10 scores compared with the reference year and should not be interpreted as absolute temporal trends. Unadjusted mean CESD-10 scores fluctuated across the nine survey waves, ranging from 1.481 in 2006 to 1.935 in 2014, with no consistent temporal pattern (Supplementary Table S1). These estimates are descriptive and were not adjusted for participant characteristics. Overall, greater subjective chewing difficulty was consistently associated with higher depressive symptom levels across repeated observations in both men and women, regardless of denture status.

Formal interaction analyses were also conducted to examine whether sex modified the associations of denture use and subjective chewing difficulty with depressive symptoms. The sex × denture use interaction was statistically significant [exp(B) = 1.064, 95% CI: 1.043–1.085, *p* < 0.001], indicating that the association between denture use and depressive symptoms differed by sex.

Significant interactions between sex and subjective chewing difficulty were also observed among both denture users and non-denture users. Among denture users, the interaction terms across chewing difficulty categories were statistically significant, and corresponding interactions were also observed among non-denture users. These findings indicate that sex modified the association between subjective chewing difficulty and depressive symptoms among both denture users and non-denture users. The exponentiated interaction estimates, 95% confidence intervals, and *p*-values are presented in Table 5.

In the sensitivity analyses using the three-level chewing difficulty classification, poor chewing ability remained significantly associated with higher expected mean CESD-10 scores among both men and women, regardless of denture status (Supplementary Table S2). The corresponding sex × chewing difficulty interaction estimates are presented in Supplementary Table S3.

## 4. Discussion

Considering the rapidly aging population in Korea, maintaining oral function and psychological well-being among middle-aged and older adults is increasingly important [24,25]. Tooth loss and impaired masticatory function are associated with poorer oral health-related quality of life and overall quality of life [26]. In the present study, greater subjective chewing difficulty was consistently associated with higher depressive symptom levels across repeated observations among both men and women and among both denture users and non-denture users. This finding is consistent with previous Korean and Japanese studies reporting associations between chewing difficulty, poor oral health, and depressive symptoms [27,28]. Difficulties in chewing, speaking, and smiling may also partially explain the relationship between tooth loss and depression [29].

The relationship between chewing difficulty and depressive symptoms may be bidirectional. Previous studies have suggested that recurrent depression may predict subsequent chewing difficulty, whereas chewing difficulty may mediate the association between edentulism and depressive symptoms [30,31]. In the present study, denture use, subjective chewing difficulty, and depressive symptoms were assessed contemporaneously within each survey wave. Therefore, although the GEE models accounted for repeated observations over time, the findings represent population-averaged associations and do not establish the temporal ordering or causal direction of these relationships.

Denture use was associated with a modestly higher expected mean CESD-10 score among women but not among men. Formal interaction analysis further indicated that the association between denture use and depressive symptoms differed by sex. Nevertheless, the magnitude of the denture use association was relatively small, whereas severe subjective chewing difficulty showed more pronounced associations with depressive symptoms in the stratified analyses. Given the large sample size and repeated observations, statistical significance should not be interpreted as equivalent to clinical importance.

Denture use itself may represent several underlying oral health conditions. Although dentures are intended to restore lost oral function, their use may also reflect extensive tooth loss and accumulated oral disease [32]. Conversely, prosthetic rehabilitation may have favorable psychosocial consequences when it adequately restores function [33]. Thus, denture status alone is unlikely to fully explain depressive symptoms. Denture fit, retention, pain, satisfaction, remaining natural teeth, and the extent of functional recovery may also be important.

The significant sex × subjective chewing difficulty interactions observed among both denture users and non-denture users further indicate that the pattern of association between chewing difficulty and depressive symptoms differed by sex. However, because the direction and magnitude of the interaction estimates varied across chewing difficulty categories, these findings should be interpreted as a sex-dependent pattern rather than as evidence of a uniformly stronger association among women.

The denture status-specific chewing questions should also be considered when interpreting these findings. Among denture users, subjective chewing difficulty reflects perceived chewing performance while using a prosthesis and may therefore be influenced by denture fit, retention, pain, and prosthetic rehabilitation. Among non-denture users, the measure more directly reflects natural or residual masticatory capacity. Consequently, identical response categories do not necessarily represent equivalent underlying oral-function states across the two denture status groups, and direct comparisons between denture users and non-denture users should therefore be made cautiously.

Chewing difficulty may be associated with mental health through nutritional, functional, and psychosocial pathways. Reduced masticatory ability has been associated with malnutrition, sarcopenia, frailty, and functional disability [34,35,36]. Chewing difficulty may also restrict food choices, reduce enjoyment of eating, and limit participation in shared meals and social activities. Because associations between eating alone and depression may vary according to sex and living arrangement, social participation and support should be considered alongside nutritional and physical factors [37].

Oral health may also reflect broader health vulnerability associated with long-term outcomes. Previous studies have reported higher mortality among individuals with severe tooth loss, edentulism, or poor self-rated oral health, whereas denture use may partly attenuate such risk [23,38,39]. These associations should not be interpreted as indicating a direct effect of oral status on mortality. Rather, oral health may reflect broader nutritional, socioeconomic, behavioral, and chronic disease-related vulnerabilities.

### 4.1. Clinical Implications

The present findings indicate that subjective chewing difficulty was consistently associated with depressive symptom levels across repeated observations, whereas the association of denture use with depressive symptoms was comparatively small. Assessment of middle-aged and older adults may therefore benefit from evaluating perceived chewing function in addition to recording denture status alone. Food-related functional limitations and depressive symptoms may also warrant consideration in individuals reporting substantial chewing difficulty [27,28,40].

Among denture users, clinical assessment may additionally include denture fit, denture-related pain, retention, satisfaction, and the extent to which prosthetic treatment restores chewing function [33]. Because subjective chewing difficulty does not necessarily correspond directly to objective masticatory performance, objective assessments may also be considered when clinically indicated [41,42]. The significant interaction findings suggest that sex may be relevant when interpreting the associations of denture use and chewing difficulty with depressive symptoms. However, the observed sex-dependent pattern does not justify assuming that these associations are uniformly stronger in one sex [43]. Rather, oral and mental health assessments should consider individual functional, nutritional, psychosocial, and health-related circumstances.

These findings do not demonstrate that improving chewing function will reduce depressive symptoms. Prospective and intervention studies are needed to determine whether oral rehabilitation and improvements in masticatory function contribute to subsequent mental health outcomes. Integrated approaches involving oral healthcare, nutritional support, social participation, and mental health services may therefore warrant further evaluation [36,37].

### 4.2. Limitations and Future Research

This study has several limitations. First, denture use, subjective chewing difficulty, and depressive symptoms were assessed contemporaneously at each survey wave. Although GEE models appropriately account for correlations among repeated observations and provide population-averaged estimates, the present analyses do not establish temporal ordering or causal direction. Reverse or bidirectional relationships therefore remain possible.

Second, denture use and chewing difficulty were self-reported and may have been affected by recall or reporting bias. Moreover, the denture status-specific chewing questions represent different functional contexts: prosthesis-assisted chewing among denture users and natural or residual chewing capacity among non-denture users. Therefore, direct comparisons of identically labeled chewing difficulty categories across denture status groups should be interpreted cautiously. Third, detailed clinical oral health information, including the number of remaining natural teeth, edentulous status, functional tooth units, occlusal status, denture type and fit, bite force, tongue pressure, and objective masticatory performance, was not consistently available across KLoSA waves [41,42]. Residual confounding by these unmeasured oral health factors therefore cannot be excluded.

Fourth, attrition due to death, declining health, non-response, and loss to follow-up occurred over the 16-year observation period. If attrition was associated with both oral function and depressive symptoms, informative attrition may have influenced the estimates. Fifth, some subjective chewing difficulty categories contained relatively few observations, particularly among denture users, which may have reduced the precision of subgroup estimates. To assess the robustness of the findings to this issue, a sensitivity analysis was conducted using a three-level chewing difficulty variable (Good, Moderate, and Poor), with the two most favorable and the two most severe response categories combined, respectively. Sixth, the GEE analyses of repeated observations were conducted without incorporating survey weights or strata. Although KLoSA was designed as a nationally representative panel survey, the present estimates should therefore be interpreted as unweighted population-averaged associations within the analytic cohort rather than as nationally weighted population estimates.

GEE models estimate population-averaged associations and do not characterize individual trajectories. Future studies using lagged, cross-lagged, mixed-effects, latent growth, or other longitudinal approaches, together with objective oral health assessments, are needed to clarify temporal direction, individual changes, and potential mechanisms linking masticatory function and depressive symptoms. Further research should also examine psychosocial factors such as denture satisfaction, social eating, social support, social isolation, and health management behaviors, and evaluate whether integrated oral health, nutritional, social, and mental health interventions improve long-term outcomes.

## 5. Conclusions

Across repeated observations from nine waves of KLoSA, greater subjective chewing difficulty was consistently associated with higher depressive symptom levels among both men and women and among both denture users and non-denture users. Sex modified the associations of denture use and subjective chewing difficulty with depressive symptoms; the association of denture use with depressive symptoms was observed among women but not among men, while the associations between subjective chewing difficulty and depressive symptoms showed sex-dependent patterns.

These findings suggest that perceived chewing function may provide additional information beyond denture status when assessing oral health-related vulnerability associated with depressive symptoms. However, temporal direction and causality cannot be established from the present analyses.

## Figures and Tables

**Table 1:** Depressive symptoms according to the sociodemographic and health-related characteristics at baseline (2006).

Variable	Depressive Symptoms								
	Total			Male			Female		
	n (%)	Mean ± SD	*p*-Value	n (%)	Mean ± SD	*p*-Value	n (%)	Mean ± SD	*p*-Value
Age, y			<0.001			0.132			<0.001
≤54	3265 (32.2)	1.19 ± 1.38		1433 (32.4)	1.15 ± 1.25		1832 (32.0)	1.23 ± 1.47	
55–64	2767 (27.3)	1.36 ± 1.59		1264 (28.6)	1.28 ± 1.55		1503 (26.3)	1.42 ± 1.61	
65–74	2655 (26.2)	1.63 ± 1.97		1201 (27.2)	1.43 ± 1.72		1454 (25.4)	1.80 ± 2.14	
≥75	1450 (14.3)	2.10 ± 2.44		521 (11.8)	1.86 ± 2.29		929 (16.3)	2.23 ± 2.51	
Education level			<0.001			0.083			<0.001
Elementary school or lower	4747 (46.8)	1.81 ± 2.07		1402 (31.7)	1.63 ± 1.89		3345 (58.5)	1.89 ± 2.13	
Middle school	1643 (16.2)	1.28 ± 1.58		752 (17.0)	1.33 ± 1.59		891 (15.6)	1.24 ± 1.58	
High school	2694 (26.6)	1.16 ± 1.45		1488 (33.7)	1.18 ± 1.45		1206 (21.1)	1.13 ± 1.45	
College or higher	1053 (10.4)	1.14 ± 1.37		777 (17.6)	1.16 ± 1.41		276 (4.8)	1.09 ± 1.26	
Marital status			<0.001			<0.001			<0.001
Married	7907 (78.0)	1.32 ± 1.58		4064 (92.0)	1.27 ± 1.51		3843 (67.2)	1.38 ± 1.65	
Separated or divorced	2144 (21.2)	2.08 ± 2.36		305 (6.9)	2.39 ± 2.50		1839 (32.2)	2.02 ± 2.33	
Never married	86 (0.8)	1.50 ± 2.14		50 (1.1)	1.38 ± 2.24		36 (0.6)	1.67 ± 2.01	
Activity limitation			<0.001			<0.001			<0.001
Yes	3448 (34.0)	2.19 ± 2.39		1205 (27.3)	2.04 ± 2.30		2243 (39.2)	2.27 ± 2.44	
No	6689 (66.0)	1.12 ± 1.26		3214 (72.7)	1.09 ± 1.20		3475 (60.8)	1.15 ± 1.32	
Alcohol consumption			0.208			0.214			<0.001
Yes	3786 (37.3)	1.36 ± 1.57		2760 (62.5)	1.25 ± 1.45		1026 (17.9)	1.65 ± 1.83	
No	6351 (62.7)	1.56 ± 1.93		1659 (37.5)	1.51 ± 1.89		4692 (82.1)	1.57 ± 1.94	
Health insurance			<0.001			<0.001			<0.001
NHI	9516 (93.9)	1.40 ± 1.70		4175 (94.5)	1.28 ± 1.54		5341 (93.4)	1.50 ± 1.81	
Medical aid	621 (6.1)	2.68 ± 2.69		244 (5.5)	2.46 ± 2.55		377 (6.6)	2.81 ± 2.77	
Number of chronic diseases *			<0.001			<0.001			<0.001
0	5253 (51.8)	1.21 ± 1.44		2466 (55.8)	1.17 ± 1.34		2787 (48.7)	1.26 ± 1.52	
1	2921 (28.8)	1.56 ± 1.87		1205 (27.3)	1.35 ± 1.62		1716 (30.0)	1.72 ± 2.01	
≥2	1963 (19.4)	2.07 ± 2.35		748 (16.9)	1.93 ± 2.26		1215 (21.3)	2.17 ± 2.39	
Total	10,137 (100.0)	1.48 ± 1.80		4419 (100.0)	1.34 ± 1.63		5718 (100.0)	1.59 ± 1.92	

Note. * Includes hypertension, diabetes, cancer, chronic obstructive pulmonary disease, liver disease, cardiovascular disease, cerebrovascular disease, psychiatric disorders, and arthritis. The *p*-values were calculated with the independent *t*-test or one-way analysis of variance test at α = 0.05. NHI, National Health Insurance; SD, standard deviation.

**Table 2:** Differences in subjective chewing difficulty and depressive symptoms by sex and denture status (2006).

Variable	Depressive Symptoms								
	Total			Male			Female		
	n (%)	Mean ± SD	*p*-Value	n (%)	Mean ± SD	*p*-Value	n (%)	Mean ± SD	*p*-Value
Denture use			<0.001			0.813			<0.001
Yes	2451 (24.2)	1.78 ± 2.10		987 (22.3)	1.57 ± 1.87		1464 (25.6)	1.92 ± 2.23	
No	7686 (75.8)	1.39 ± 1.69		3432 (77.7)	1.28 ± 1.55		4254 (74.4)	1.47 ± 1.79	
Chewing difficulty with dentures			<0.001			<0.001			<0.001
Very well	31 (1.3)	1.94 ± 1.29		15 (1.5)	2.20 ± 1.01		16 (1.1)	1.69 ± 1.49	
Well	663 (27.1)	1.48 ± 1.67		295 (29.9)	1.41 ± 1.55		368 (25.1)	1.53 ± 1.76	
Moderately	888 (36.2)	1.50 ± 1.99		368 (37.3)	1.30 ± 1.70		520 (35.5)	1.64 ± 2.17	
With difficulty	809 (33.0)	2.25 ± 2.37		291 (29.5)	1.99 ± 2.20		518 (35.4)	2.39 ± 2.44	
Unable to chew at all	60 (2.4)	2.93 ± 2.91		18 (1.8)	2.50 ± 3.15		42 (2.9)	3.12 ± 2.81	
Chewing difficulty without dentures			<0.001			<0.001			<0.001
Very well	486 (6.3)	1.36 ± 1.27		228 (6.7)	1.26 ± 1.17		258 (6.1)	1.44 ± 1.35	
Well	3929 (51.1)	1.15 ± 1.38		1851 (53.9)	1.10 ± 1.29		2078 (48.8)	1.19 ± 1.47	
Moderately	2003 (26.1)	1.37 ± 1.71		830 (24.2)	1.24 ± 1.61		1173 (27.6)	1.46 ± 1.77	
With difficulty	1147 (14.9)	2.08 ± 2.25		474 (13.8)	1.81 ± 1.99		673 (15.8)	2.26 ± 2.40	
Unable to chew at all	121 (1.6)	3.06 ± 2.65		49 (1.4)	3.55 ± 2.95		72 (1.7)	2.72 ± 2.39	
Total	10,137 (100.0)	1.48 ± 1.80		4419 (100.0)	1.34 ± 1.63		5718 (100.0)	1.59 ± 1.92	

Note. The *p*-values were calculated with the independent *t*-test or one-way analysis of variance test at α = 0.05. SD, standard deviation.

**Table 3:** Association between denture use and depressive symptoms.

Variable	Depressive Symptoms					
	Total		Male		Female	
	exp(B) (95% CI)	*p*-Value	exp(B) (95% CI)	*p*-Value	exp(B) (95% CI)	*p*-Value
Denture use						
Yes	1.049 (1.031–1.067)	<0.001	1.018 (0.991–1.045)	0.203	1.068 (1.044–1.092)	<0.001
No	1		1		1	
Age, y						
≤54	1		1		1	
55–64	1.060 (1.028–1.092)	<0.001	1.016 (0.973–1.061)	0.470	1.093 (1.049–1.140)	<0.001
65–74	1.117 (1.083–1.152)	<0.001	1.064 (1.018–1.112)	0.006	1.164 (1.115–1.216)	<0.001
≥75	1.206 (1.166–1.247)	<0.001	1.114 (1.062–1.169)	<0.001	1.290 (1.230–1.352)	<0.001
Education level						
Elementary school or lower	1.054 (1.022–1.087)	0.001	1.065 (1.027–1.105)	0.001	1.034 (0.978–1.093)	0.241
Middle school	1.006 (0.973–1.039)	0.727	1.044 (1.004–1.086)	0.031	0.968 (0.913–1.027)	0.281
High school	1.023 (0.993–1.054)	0.138	1.042 (1.007–1.078)	0.019	1.000 (0.945–1.059)	0.994
College or higher	1		1		1	
Marital status						
Married	1		1		1	
Separated or divorced	1.131 (1.111–1.151)	<0.001	1.244 (1.204–1.285)	<0.001	1.082 (1.058–1.106)	<0.001
Never married	1.167 (1.087–1.254)	<0.001	1.068 (0.970–1.177)	0.181	1.266 (1.142–1.405)	<0.001
Activity limitation						
Yes	1.355 (1.333–1.377)	<0.001	1.325 (1.293–1.358)	<0.001	1.370 (1.340–1.399)	<0.001
No	1		1		1	
Alcohol consumption						
Yes	1		1		1	
No	1.064 (1.045–1.084)	<0.001	1.116 (1.090–1.142)	<0.001	1.003 (0.975–1.031)	0.849
Health insurance						
NHI	1		1		1	
Medical aid	1.223 (1.194–1.252)	<0.001	1.199 (1.153–1.247)	<0.001	1.230 (1.193–1.268)	<0.001
Number of chronic diseases *						
0	1		1		1	
1	1.161 (1.138–1.184)	<0.001	1.150 (1.115–1.186)	<0.001	1.161 (1.131–1.193)	<0.001
≥2	1.357 (1.314–1.401)	<0.001	1.442 (1.378–1.509)	<0.001	1.295 (1.238–1.354)	<0.001
Year						
2006	0.820 (0.790–0.850)	<0.001	0.761 (0.721–0.804)	<0.001	0.868 (0.826–0.911)	<0.001
2008	1.159 (1.122–1.196)	<0.001	1.050 (1.000–1.103)	0.051	1.232 (1.181–1.286)	<0.001
2010	1.112 (1.076–1.149)	<0.001	1.040 (0.989–1.093)	0.123	1.160 (1.110–1.213)	<0.001
2012	1.006 (0.972–1.041)	0.720	0.930 (0.882–0.979)	0.006	1.058 (1.011–1.108)	0.014
2014	1.120 (1.085–1.157)	<0.001	1.104 (1.053–1.158)	<0.001	1.132 (1.084–1.183)	<0.001
2016	1.061 (1.027–1.097)	<0.001	1.049 (0.999–1.102)	0.056	1.070 (1.023–1.119)	0.003
2018	1.036 (1.001–1.071)	0.044	1.027 (0.976–1.080)	0.304	1.041 (0.995–1.090)	0.084
2020	1.043 (1.008–1.080)	0.015	1.062 (1.010–1.117)	0.018	1.032 (0.985–1.081)	0.189
2022	1		1		1	

Note. The *p*-values were calculated using a generalized estimating equation model at α = 0.05. Models were mutually adjusted for age, education level, marital status, activity limitation, alcohol consumption, health insurance type, the number of chronic conditions, and survey year, as applicable. * Includes hypertension, diabetes, cancer, chronic obstructive pulmonary disease, liver disease, cardiovascular disease, cerebrovascular disease, psychiatric disorders, and arthritis. exp(B), exponentiated regression coefficient, interpreted as the ratio of expected mean CESD-10 scores relative to the reference category; CI, confidence interval; NHI, National Health Insurance.

**Table 4:** Association between subjective chewing difficulty and depressive symptoms by sex and denture status.

Variable	Depressive Symptoms							
	Model 1: Denture Users				Model 2: Non-Denture Users			
	Male		Female		Male		Female	
	exp(B) (95% CI)	*p*-Value	exp(B) (95% CI)	*p*-Value	exp(B) (95% CI)	*p*-Value	exp(B) (95% CI)	*p*-Value
Chewing difficulty with dentures								
Very well	1		1					
Well	0.924 (0.692–1.233)	0.590	0.928 (0.643–1.340)	0.690				
Moderately	0.962 (0.724–1.280)	0.791	0.989 (0.687–1.424)	0.954				
With difficulty	1.284 (0.966–1.705)	0.085	1.385 (0.962–1.992)	0.080				
Unable to chew at all	1.911 (1.418–2.576)	<0.001	1.875 (1.297–2.712)	<0.001				
Chewing difficulty without dentures								
Very well					1		1	
Well					0.960 (0.890–1.035)	0.284	0.945 (0.875–1.020)	0.144
Moderately					1.031 (0.955–1.112)	0.442	1.074 (0.994–1.160)	0.069
With difficulty					1.322 (1.220–1.433)	<0.001	1.351 (1.247–1.463)	<0.001
Unable to chew at all					1.553 (1.403–1.720)	<0.001	1.648 (1.500–1.811)	<0.001
Age, y								
≤54	1		1		1		1	
55–64	0.958 (0.775–1.185)	0.693	0.970 (0.776–1.212)	0.788	0.991 (0.950–1.035)	0.698	1.058 (1.016–1.101)	0.006
65–74	0.946 (0.770–1.162)	0.597	0.994 (0.801–1.233)	0.955	1.032 (0.986–1.080)	0.178	1.116 (1.068–1.166)	<0.001
≥75	1.031 (0.839–1.267)	0.770	1.113 (0.897–1.381)	0.332	1.010 (0.959–1.064)	0.695	1.146 (1.090–1.205)	<0.001
Education level								
Elementary school or lower	0.978 (0.893–1.071)	0.628	0.894 (0.705–1.133)	0.355	1.043 (1.003–1.086)	0.036	1.013 (0.960–1.070)	0.630
Middle school	0.995 (0.898–1.101)	0.915	0.782 (0.611–1.002)	0.052	1.028 (0.986–1.072)	0.193	0.998 (0.943–1.056)	0.939
High school	1.025 (0.931–1.129)	0.615	0.852 (0.663–1.097)	0.214	1.039 (1.003–1.076)	0.034	1.008 (0.955–1.064)	0.763
College or higher	1		1		1		1	
Marital status								
Married	1		1		1		1	
Separated or divorced	1.179 (1.108–1.255)	<0.001	1.097 (1.053–1.144)	<0.001	1.277 (1.231–1.325)	<0.001	1.048 (1.021–1.076)	<0.001
Never married	0.982 (0.677–1.424)	0.923	1.088 (0.841–1.406)	0.522	1.070 (0.974–1.176)	0.159	1.302 (1.170–1.449)	<0.001
Activity limitation								
Yes	1.330 (1.262–1.402)	<0.001	1.411 (1.347–1.477)	<0.001	1.266 (1.231–1.302)	<0.001	1.269 (1.238–1.300)	<0.001
No	1		1		1		1	
Alcohol consumption								
Yes	1		1		1		1	
No	1.153 (1.096–1.213)	<0.001	1.015 (0.956–1.079)	0.622	1.095 (1.067–1.123)	<0.001	1.003 (0.973–1.034)	0.851
Health insurance								
NHI	1		1		1		1	
Medical aid	1.044 (0.964–1.130)	0.290	1.169 (1.109–1.232)	<0.001	1.234 (1.181–1.290)	<0.001	1.205 (1.161–1.251)	<0.001
Number of chronic diseases *								
0	1		1		1		1	
1	1.250 (1.182–1.321)	<0.001	1.138 (1.085–1.194)	<0.001	1.092 (1.053–1.132)	<0.001	1.157 (1.121–1.194)	<0.001
≥2	1.564 (1.442–1.697)	<0.001	1.118 (1.028–1.216)	0.009	1.372 (1.299–1.448)	<0.001	1.372 (1.302–1.445)	<0.001
Year								
2006	0.779 (0.691–0.878)	<0.001	0.938 (0.848–1.038)	0.219	0.713 (0.671–0.757)	<0.001	0.791 (0.748–0.836)	<0.001
2008	1.136 (1.019–1.265)	0.021	1.326 (1.214–1.449)	<0.001	0.950 (0.899–1.003)	0.064	1.112 (1.060–1.167)	<0.001
2010	1.129 (1.012–1.259)	0.030	1.291 (1.180–1.412)	<0.001	0.962 (0.910–1.016)	0.163	1.046 (0.995–1.099)	0.076
2012	1.182 (1.062–1.316)	0.002	1.287 (1.176–1.407)	<0.001	0.826 (0.778–0.877)	<0.001	0.938 (0.890–0.989)	0.017
2014	1.100 (0.982–1.232)	0.100	1.165 (1.061–1.279)	0.001	1.062 (1.009–1.118)	0.021	1.083 (1.033–1.137)	0.001
2016	1.146 (1.027–1.280)	0.015	1.102 (1.003–1.212)	0.044	0.990 (0.938–1.045)	0.719	1.035 (0.985–1.087)	0.173
2018	1.064 (0.953–1.189)	0.270	1.060 (0.964–1.166)	0.229	0.998 (0.944–1.054)	0.932	1.016 (0.966–1.069)	0.540
2020	1.043 (0.929–1.171)	0.473	1.008 (0.911–1.115)	0.882	1.034 (0.979–1.091)	0.228	1.012 (0.963–1.065)	0.635
2022	1		1		1		1	

Note. The *p*-values were calculated using a generalized estimating equation model at α = 0.05. Models were mutually adjusted for age, education level, marital status, activity limitation, alcohol consumption, health insurance type, the number of chronic conditions, and survey year, as applicable. * Hypertension, diabetes, cancer, chronic obstructive pulmonary disease, liver disease, cardiovascular disease, cerebrovascular disease, psychiatric disorders, and arthritis. exp(B), exponentiated regression coefficient, interpreted as the ratio of expected mean CESD-10 scores relative to the reference category; CI, confidence interval; NHI, National Health Insurance.

**Table 5:** Interaction effects of sex with denture use and subjective chewing difficulty in relation to depressive symptoms.

Analysis	Interaction Term	exp(B)	95% CI	*p*-Value
Denture use × sex	Denture use (Yes) × Female	1.064	1.043–1.085	<0.001
Chewing difficulty × sex among denture users	Very well × Male (Ref.)	1		
	Well × Female	0.829	0.775–0.887	<0.001
	Moderately × Female	0.878	0.838–0.921	<0.001
	With difficulty × Female	1.230	1.179–1.283	<0.001
	Unable to chew at all × Female	1.675	1.560–1.799	<0.001
Chewing difficulty × sex among non-denture users	Very well × Male (Ref.)	1		
	Well × Female	0.877	0.856–0.900	<0.001
	Moderately × Female	0.962	0.940–0.984	0.001
	With difficulty × Female	1.188	1.160–1.217	<0.001
	Unable to chew at all × Female	1.383	1.338–1.430	<0.001

Note. Interaction effects were estimated using generalized estimating equations (GEEs) with a normal distribution and log link function. The sex × denture use interaction was estimated in the pooled sample, whereas sex × subjective chewing difficulty interactions were estimated separately among denture users and non-denture users. Models were adjusted for the same covariates as the main GEE analyses. Male sex, non-use of dentures, and “very well” chewing ability were used as the reference categories, as applicable. exp(B), exponentiated interaction coefficient, representing the relative difference in expected mean CESD-10 ratios between sex groups; CI, confidence interval; GEE, generalized estimating equations; Ref., reference category. Statistical significance was set at α = 0.05.

## Data Availability

The KLoSA data used in this study are publicly available from the Korean Longitudinal Study of Aging database administered by the Korea Employment Information Service. The SAS analytic code and definitions of derived variables are available from the corresponding author upon reasonable request.

## Supplementary Materials

The following supporting information can be downloaded at: https://www.mdpi.com/article/10.3390/healthcare14182912/s1; Table S1: Wave-specific participant numbers and unadjusted mean CESD-10 scores with 95% CIs across nine survey waves; Table S2: Sensitivity analysis of the association between subjective chewing difficulty and depressive symptoms after recategorizing chewing difficulty into three levels; Table S3: Interaction effects of sex and subjective chewing difficulty on depressive symptoms after recategorizing chewing difficulty into three levels, stratified by denture status.

## Abbreviations

The following abbreviations are used in this manuscript:

KLoSA	Korean Longitudinal Study of Aging
CESD-10	10-item Center for Epidemiologic Studies Depression Scale
GEE	generalized estimating equation
CIs	confidence intervals
IRB	Institutional Review Board
